# Telehealth Usage Among Low-Income Racial and Ethnic Minority Populations During the COVID-19 Pandemic: Retrospective Observational Study

**DOI:** 10.2196/43604

**Published:** 2023-05-12

**Authors:** Cynthia Williams, Di Shang

**Affiliations:** 1 School of Global Health Management and Informatics University of Central Florida Orlando, FL United States; 2 Department of Management University of North Florida Jacksonville, FL United States

**Keywords:** COVID-19, telehealth, health equity, minority health, low income, healthcare access, pandemic, USA, United States, healthcare system, health care, risk, minority, database, utilization, education

## Abstract

**Background:**

Despite considerable efforts to encourage telehealth use during the COVID-19 pandemic, we witnessed a potential widening of health inequities that may continue to plague the US health care system unless we mitigate modifiable risk factors.

**Objective:**

This study aimed to examine the hypothesis that there are systemic differences in telehealth usage among people who live at or below 200% of the federal poverty level. Factors that we consider are age, gender, race, ethnicity, education, employment status, household size, and income.

**Methods:**

A retrospective observational study was performed using the COVID-19 Research Database to analyze factors contributing to telehealth inequities. The study period ranged from March 2020 to April 2021. The Office Ally database provided US claims data from 100 million unique patients and 3.4 billion claims. The Analytics IQ PeopleCore Consumer database is nationally representative of 242.5 million US adults aged 19 years and older. We analyzed medical claims to investigate the influence of demographic and socioeconomic factors on telehealth usage among the low-income racial and ethnic minority populations. We conducted a multiple logistic regression analysis to determine the odds of patients in diverse groups using telehealth during the study period.

**Results:**

Among 2,850,831 unique patients, nearly 60% of them were female, 75% of them had a high school education or less, 49% of them were unemployed, and 62% of them identified as non-Hispanic White. Our results suggest that 9.84% of the patients had ≥1 telehealth claims during the study period. Asian (odds ratio [OR] 1.569, 95% CI 1.528-1.611, *P*<.001) and Hispanic (OR 1.612, 95% CI 1.596-1.628, *P*<.001) patients were more likely to use telehealth than non-Hispanic White and -Black patients. Patients who were employed full-time were 15% (OR 1.148, 95% CI 1.133-1.164, *P*<.001) more likely to use telehealth than unemployed patients. Patients who identified as male were 12% (OR 0.875, 95% CI 0.867-0.883, *P*<.001) less likely to use telehealth than those who identified as female. Patients with high school education or less were 5% (OR 0.953, 95% CI 0.944-0.962, *P*<.001) less likely to use telehealth than those with a bachelor’s degree or higher. Patients in the 18-44–year age group were 32% (OR 1.324, 95% CI 1.304-1.345, *P*<.001) more likely to use telehealth than those in the ≥65-year age group.

**Conclusions:**

Factors that impact telehealth usage include age, gender, race, education, employment status, and income. While low-income racial and ethnic minority communities are at greater risk for health inequities among this group, Hispanic communities are more likely to use telehealth, and non-Hispanic Black patients continue to demonstrate telehealth inequity. Gender, age, and household income contribute to health inequities across gradients of poverty. Strategies to improve health use should consider characteristics of subgroups, as people do not experience poverty equally.

## Introduction

The COVID-19 pandemic offered a glimpse of what could occur if inequities in telehealth usage are not alleviated. The global health emergency led to significant actions by federal and state agencies to mitigate the spread of the virulent contagion. Simultaneously, there were considerable efforts to provide safe access to needed health care services, while minimizing in-person contact among health providers and patients. Public health officials supported measures to decrease telehealth restrictions and increase reimbursement for telehealth services such as store and forward services, remote patient monitoring, and audio only (telephone) services. The Centers for Disease Control and Prevention estimated that telehealth visits increased by 154% in March 2020 when compared to the same time frame in 2019 [1]. There was a 20-fold increase in telehealth visits and a 50% decrease in office-based visits; however, not all communities experienced similar telehealth usage trends [2]. While the removal of the regulatory barriers increased health care access among many individuals, it did not address the barriers experienced among low-income racial and ethnic minority individuals. While much of the research examines telehealth usage across socioeconomic statuses, this study focuses on usage among low-income groups. By focusing on this population, we aim to understand specific usage patterns that may help inform strategies to mitigate telehealth inequity.

Poverty has a significant impact on health access, usage, and outcomes. The intersectionality of poverty and race magnifies health inequities in the health care and public health systems [3,4]. Non-Hispanic Black individuals have shorter life expectancies, experience greater financial hardships, have an increased prevalence of chronic diseases, and they lack access to education and other economic opportunities [5]. Other racial groups, particularly Hispanic and American Indian communities are more likely to have lower incomes and fewer educational and economic opportunities. The interconnected nature of socioeconomic status and demographic status is central to almost every aspect of the health inequity issue, including telehealth. Previous studies suggested that low-income racial and ethnic minority groups are less likely to use technology for health-related purposes [6-9]. The requirements of telehealth could be equal to or greater than those of traditional in-person visits. Telehealth requires a sufficient device, stable internet connection, private space, and digital literacy skills, which may not be adequate among this group [10]. It is imperative that we examine telehealth usage among people in low-income racial and ethnic minority positions.

While many studies examine people across socioeconomic statuses and make conclusions about people who live in poverty, this study investigates telehealth use only among people who experience poverty, as these experiences are not equal. In this study, we use federal-level guidelines to conceptualize poverty [11]. We hypothesize that there are systemic differences in telehealth usage among people who live at or below 200% of the federal poverty level. We hypothesize that telehealth usage is positively associated with education and employment status, while it is negatively associated with age. We hypothesize that women use telehealth more than men. We also hypothesize that household income is positively associated with telehealth use when controlling for household size. Findings from this study will inform strategies across various gradients of poverty. By understanding the factors that are associated with telehealth use, we recommend strategies to promote the use of telehealth services among people who experience poverty in the United States.

## Methods

### Data Source

The COVID-19 Research Database Consortium provided data for the study. The consortium, facilitated by Datavant, is a private and public partnership across industries in the United States to facilitate data sharing and promote public health research. The Consortium provided access to Office Ally and Analytics IQ PeopleCore Consumer linked databases. The Office Ally database provided deidentified US claims data from 100 million unique patients and 3.4 billion medical claims. The Analytics IQ PeopleCore consumer database is a nationally representative database of 242.5 million US adults aged 19 years and older. Analytics IQ PeopleCore Consumer data provided deidentified patient-level data including health characteristics, medical care, and social determinants of health to help decision makers better understand their patients. With the linked identifiers (common tokens) provided by COVID-19 Research Database, we combined the Office Ally claims data with Analytics IQ PeopleCore Consumer data, which enabled us to retrieve patient-related information and examine telehealth usage across demographic and socioeconomic indicators. Telehealth in this study was defined as a range of web-based communications including remote monitoring, telephone calls, and videoconferencing.

### Ethical Considerations

The COVID-19 Research Database was established in compliance with regulatory standards to protect patient privacy. The COVID-19 Research Database received a waiver of patient consent by the Western Institutional Review Board for the use of Health Insurance Portability and Accountability Act (HIPAA)–certified deidentified data on April 20, 2020. Exemption status was granted by the Western Institutional Review Board for HIPAA-limited data sets and non–HIPAA-covered data on May 14, 2020. This exemption covers all research performed in the COVID-19 Research Database. In addition, researchers with approved study proposals are granted access only to specific data sets that are necessary to answer their research questions. Only deidentified and limited data sets are made available through the database and certified before access was granted. Individual project institutional board approval was not needed.

### Study Sample

The study period was from March 2020 to April 2021. To investigate telehealth usage in low-income populations, data were retrieved from claim records of 2,850,831 patients whose household incomes were at or below 200% of the federal poverty level. Telehealth claims were identified by screening for current procedural terminology modifier codes 95, GT, and GQ. The current procedural terminology is a medical code set that uses a uniform language for coding and reporting health services and medical procedures. The modifiers 95, GT, and GQ supplement claim forms by adding extra information about the services provided. In this case, these codes informed us that the services were delivered via telehealth.

### Statistical Analysis

The data were aggregated at the patient level to investigate telehealth usage; that is, whether a patient used telehealth during the study period. A patient with ≥1 telehealth claims during the study period was assigned a value of 1 to the dependent variable, otherwise 0. R software (The R Foundation) was used for the analysis. A multiple logistic regression analysis was used to determine the odds of using telehealth among patients in different subgroups during the study period. Categorical variables were created to divide patients into groups by demographic and socioeconomic characteristics. The total number of claims of each patient during the study period was included in the logistic regression analysis to control its potential impact on the dependent variable—telehealth usage. A *P* value of <.01 was considered statistically significant.

## Results

### Patient Characteristics

We analyzed 2,850,831 unique patients and their claim records. The results indicate that among patients in low-income positions, 9.84% of them had ≥1 telehealth claim during the study period. In comparison, among patients whose incomes are above the low-income levels (200% of the federal poverty level), 12.86% of them had ≥1 telehealth claim during the study period. The total number of claims of each patient during the study period ranged from 1 to 16 (mean 3.75, SD 3.59). Nearly 60% of participants were female, 75% of them had a high school education or less, 49% of them were unemployed, and 62% of them identified as non-Hispanic White. Patient characteristics are summarized in Table 1.

**Table 1:** Description of the patients in this study (N=2,850,831).

Characteristic		Patients, n (%)
**Telehealth usage**		
	No	2,570,252 (90.16)
	Yes	280,579 (9.84)
**Gender**		
	Female	1,696,378 (59.50)
	Male	1,154,453 (40.50)
**Age group (years)**		
	≥65	954,908 (33.50)
	45-64	1,014,759 (35.60)
	18-44	881,164 (30.91)
**Education**		
	High (bachelor’s degree or higher)	712,750 (25.00)
	Low (high school or less)	2,138,081 (75.00)
**Employment**		
	Unemployed	1,395,440 (48.95)
	Part-time	667,659 (23.42)
	Full-time	787,732 (27.63)
**Race** **and** **ethnicity**		
	Non-Hispanic White	1,768,493 (62.03)
	Asian	55,161 (1.93)
	Non-Hispanic Black	422,415 (14.82)
	Hispanic	579,641 (20.33)
	Other	25,121 (0.88)

### Telehealth Usage

As shown in Table 2, the results of our logistic regression analysis suggest that gender, age, education, race, ethnicity, and employment influence telehealth usage among people whose household incomes were at or below 200% of the federal poverty level. The *P* values suggest that Asian and Hispanic patients are more likely to use telehealth than non-Hispanic White and -Black patients; telehealth usage was not significantly different between non-Hispanic White and -Black patients. Patients who identified as male were 12% less likely to use telehealth than those who identified as female. Additionally, patients with high school or less education were 5% less likely to use telehealth than those with a bachelor’s degree or higher. Our results also suggest that patients in the age group of 18-44 years are 32% more likely to use telehealth than those in the ≥65-year age group. Meanwhile, patients with full-time employment were 15% more likely to use telehealth than those who were unemployed. The results support the hypothesis that telehealth usage is positively associated with education and employment status but negatively associated with age. The results also support the hypothesis that women use telehealth more than men.

**Figure 1:**
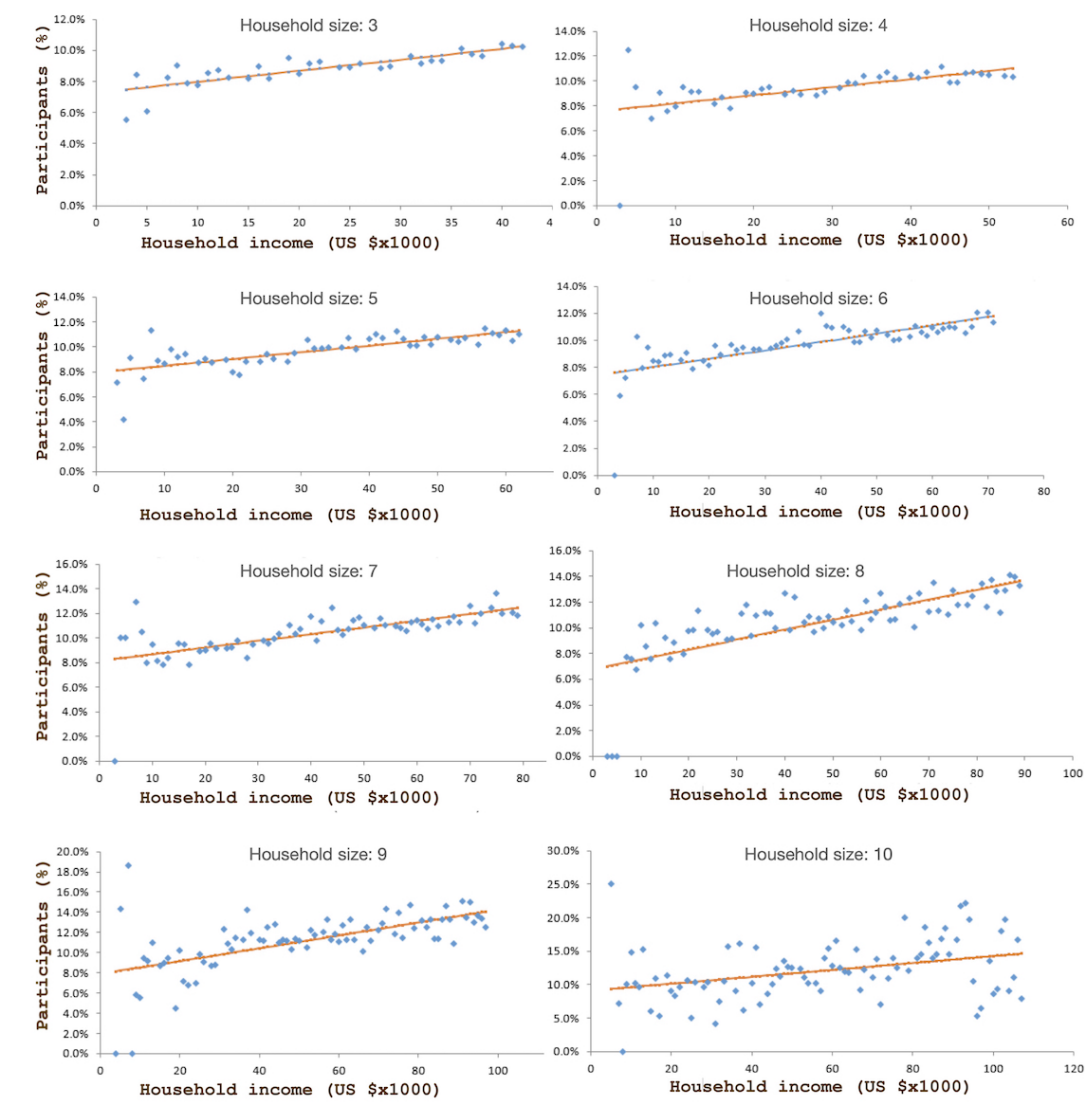
Summary of the percentage of patients having telehealth visits by household income.

We carried out a simple linear regression analysis to investigate the impact of household income on telehealth use within each household size. The dependent variable is the percentage of patients who used telehealth at each income level, and the independent variable is income level. Our linear regression analysis of the patients by household size suggests that within the low-income population, income is a contributor to telehealth usage in households of >2 people. Among 1-2–person households, the association of household income and telehealth usage is insignificant (*P*=.86 and *P*=.23, respectively). As shown in Figure 1, for patients in 3-10–person households, income is significantly (*P*<.001) associated with the percentage of patients using telehealth at each income level. For example, the telehealth usage of patients in 3-person households ranged from 5% (for those with a household income of US $3000) to 10% (for those with a household income of US $42,000). We validate the impact of income by performing multiple logistic regression analyses at the patient level within each household size. Our results suggest that within each household size, income is significantly (*P*<.001) and positively associated with the odds that a patient uses telehealth, accounting for the effects of age, gender, education, employment, and race. The results support the hypothesis that there are systemic differences in telehealth usage among people who live at or below 200% of the federal poverty level. Factors that impact telehealth usage include age, gender, race, ethnicity, education, employment status, household size, and income. However, among household size and income variables, our hypothesis is supported for at least 3-person households and their income. Thus, there is a positive relationship between household income and telehealth use among people who live at or below 200% of the federal poverty level—this is attributed to living in households with at least 3 people.

**Table 2:** Summary of odds ratios from logistic regression analysis.

Characteristic		Odds ratio (95% CI)	*P* value
**Age group (years; reference: ≥65 years)**			
	45-64	1.123 (1.108-1.138)	<.001
	18-44	1.324 (1.304-1.345)	<.001
**Gender (reference: female)**			
	Male	0.875 (0.867-0.883)	<.001
**Education (reference: bachelor’s degree or higher) **			
	High school or less	0.953 (0.944-0.962)	<.001
**Employment(reference: unemployed participants)**			
	Part-time	1.067 (1.053-1.081)	<.001
	Full-time	1.148 (1.133-1.164)	<.001
**Race and ethnicity(reference: non-Hispanic White)**			
	Asian	1.569 (1.528-1.611)	<.001
	Non-Hispanic Black	0.994 (0.981-1.006)	.32
	Hispanic	1.612 (1.596-1.628)	<.001
	Other	1.296 (1.242-1.352)	<.001

## Discussion

### Principal Findings

#### Household Size and Income, Employment, and Telehealth Use

Our study examined telehealth use among people whose household incomes were at or below 200% of the federal poverty level, and we found that 9.84% of the study sample used telehealth services. Previous studies have examined mean household income across all income levels and found a positive relationship between income and telehealth use [12-14]. Our study adds the gradient of household size and income with telehealth use among people who live in poverty; income and telehealth use are positively associated with living in at least a 3-person household. Our study also adds that full-time employment was a contributor to telehealth use. Other studies have suggested that insurance status, rather than employment, was a greater indicator of telehealth use [14,15]. The lack of internet and computer usage in the digital health era poses significant barriers to health care access for persons who are underemployed or unemployed.

#### Race and Telehealth Use

Among people in low-income positions, there were racial and ethnic differences, but they were different from those reported in studies that examine income more broadly. When all income levels are considered, studies suggested that telehealth use among non-Hispanic White patients was greater than that in non-Hispanic Black and Hispanic patients [12,16,17]. Among low-income populations, we found that Hispanic patients had higher odds of using telehealth services than non-Hispanic White and -Black patients. This is reasonable considering a Pew Research Center study [18] that suggested that Hispanic respondents’ broadband use over time surpassed that of non-Hispanic White and -Black respondents. Non-Hispanic Black patients had lower odds of using telehealth, but it was not significantly different from that of non-Hispanic White patients. Lee et al [19] in 2021 suggested that access to care does not equal the usage of care. When access to care increased, inequities in care usage between Hispanic and non-Hispanic White patients decreased; however, inequities in care usage between non-Hispanic White and -Black patients were similar despite an increase in access to health care services [19]. Zhang et al [12] found that at 138% below the federal poverty level, the racial and ethnic inequities in health care usage were lessened, but ethnic and racial differences were noted.

#### Other Considerations in Telehealth Use

The influence of age, gender, and education level on telehealth use is similar across gradients of poverty. Individuals older than 60 years or men are less likely to use telehealth services [20,21]. This is not surprising, given that younger adults aged 18-44 years are more engaged with technology [22]. As age increased, telehealth use decreased [20,23,24]. However, trends are promising and suggest that older adults are engaging increasingly in health-related technology [25]. Studies have noted gender differences in telehealth usage; as men are less likely to use telehealth than women, some studies postulate that women may be more inclined to use telehealth services for convenience reasons [26-28]. Our study noted that having a high school diploma or less is a significant barrier to telehealth use. This aligns with previous studies that suggested that the lack of education is a primary barrier to health care among people in low-income positions and has significant implications for literacy [3,22,29]. A lack of education promotes low health literacy, constraining the community’s ability to access and use health-related information, and increases the digital divide [30,31].

### Applications

Enhancement of digital inclusion supports a reduction in inequities by addressing issues that are specific to subgroups of people [32,33]. Education (health and digital literacy) is intricately linked with employment, income, and technology use [34,35]. Thus, education can be a tool to promote equity in telehealth, employment, and income status. In many instances, local health care and public health systems can conceptualize telehealth inequities to their specific communities and tailor support services that consider characteristics beyond income [36]. Telehealth should be appropriately positioned with support strategies to foster engagement in ways that overcome modifiable barriers and consider nonmodifiable factors such as race, ethnicity, age, and gender [37]. For example, adding broadband internet access to the public health infrastructure, providing computers or laptops, and supporting education that advocates for the use of technology for health-related reasons are just the beginning. This may be a reasonable starting point for Hispanic communities, but a different strategy may be needed for non-Hispanic Black and -White communities, men, and older adults, which consider gender, generational, and cultural perspectives to promote telehealth use.

### Limitations

While this study is not an exhaustive examination of factors that influenced telehealth use, we did consider key factors that may contribute to inequities in usage. The study used the COVID-19 Research Database and is subject to the limitations of administrative databases. In the Office Ally database, the validity of the data is reliant upon the facilities to report accurate data and code visits correctly. The Analytic IQ PeopleCore Consumer database relies on the accuracy of consumer reporting. This study did not consider contextual factors such as the availability of providers who used telehealth, residential segregation, and the lack of a racial and ethnic minority workforce. Future studies should consider these factors and the variety of cultural perspectives in communities. Focus groups of patients and providers in these and other communities may help explore additional information not captured in surveys and claims data that explicate attitudes and challenges with telehealth access and use. Future work could parse out the influence of sociodemographic characteristics on the type of visits used by this population. Such information could be used to develop community-specific programs that facilitate telehealth access either through education or access to technology equipment.

### Conclusions

Our study concludes that among people whose incomes are below the federal poverty threshold, Hispanic and Asian patients were more likely to use telehealth than non-Hispanic White and -Black patients. Patients who are employed full-time, female, aged between 18 and 44 years, and had completed a bachelor’s degree were more likely to use telehealth. Income is positively associated with telehealth usage in 3- to 10-person households. As we seek to promote telehealth usage, it is imperative that we consider the socioeconomic and demographic factors among subgroups of people who experience poverty. Due to the long-standing challenges in the US health care system, inequities have the potential to become entrenched in our society unless we take decisive action to address these challenges. By focusing on communities in low-income positions, we provide professionals and decision makers with additional insight to promote public health in an increasingly digital society. The tragic events of COVID-19 the pandemic show us that we need to bolster the public health infrastructure and take a more meaningful and targeted approach to health equity concerns.

## Abbreviations

HIPAA: Health Insurance Portability and Accountability Act
OR: odds ratio

## Data Availability

The data sets generated and/or analyzed during the current study are not publicly available due to the Consortium’s restrictions and governance policies. The database can be accessed by academic, scientific, and medical researchers from its website [38]. Potential users must register as an approved researcher and submit their proposal, including a request to access a specific database. The submitted proposal will undergo a review by the scientific steering committee and a privacy and governance review board. If approved, access will be granted to install the database environment in which researchers must conduct the analysis.

## References

[1] Koonin LM, Hoots B, Tsang CA, Leroy Z, Farris K, Jolly B, Antall P, McCabe B, Zelis CB, Tong I, Harris AM (2020). Trends in the use of telehealth during the emergence of the COVID-19 pandemic - United States, January-March 2020. MMWR Morb Mortal Wkly Rep.

[2] Kaufman J, Diliberti M (2021). Divergent and inequitable teaching and learning pathways during (and perhaps beyond) the pandemic. Key findings from the American Educator Panels Spring 2021 COVID-19 surveys. RAND Corporation.

[3] Lazar M, Davenport L (2018). Barriers to health care access for low income families: a review of literature. J Community Health Nurs.

[4] Khullar D, Chokshi DA (2018). Can better care coordination lower health care costs?. JAMA Netw Open.

[5] Manandhar M, Hawkes S, Buse K, Nosrati E, Magar V (2018). Gender, health and the 2030 agenda for sustainable development. Bull World Health Organ.

[6] Chang Ji E, Lai Alden Yuanhong, Gupta Avni, Nguyen Ann M, Berry Carolyn A, Shelley Donna R (2021). Rapid transition to telehealth and the digital divide: implications for primary care access and equity in a post-COVID era. Milbank Q.

[7] Smith CB, Bhardwaj AS (2020). Disparities in the use of telehealth during the COVID-19 pandemic. J Clin Oncol.

[8] Weber E, Miller S, Astha V, Janevic T, Benn E (2020). Characteristics of telehealth users in NYC for COVID-related care during the coronavirus pandemic. J Am Med Inform Assoc.

[9] Ramirez AV, Ojeaga M, Espinoza V, Hensler B, Honrubia V (2021). Telemedicine in minority and socioeconomically disadvantaged communities amidst COVID-19 pandemic. Otolaryngol Head Neck Surg.

[10] Saeed SA, Masters RM (2021). Disparities in health care and the digital divide. Curr Psychiatry Rep.

[11] U.S. Federal Poverty Guidelines Used to Determine Financial Eligibility for Certain Programs. HHS Poverty Guidelines for 2023. Office of the Assistant Secretary for Planning and Evaluation.

[12] Zhang D, Shi L, Han X, Li Y, Jalajel NA, Patel S, Chen Z, Chen L, Wen M, Li H, Chen B, Li J, Su D (2021). Disparities in telehealth utilization during the COVID-19 pandemic: findings from a nationally representative survey in the United States. J Telemed Telecare.

[13] Chunara R, Zhao Yuan, Chen Ji, Lawrence Katharine, Testa Paul A, Nov Oded, Mann Devin M (2021). Telemedicine and healthcare disparities: a cohort study in a large healthcare system in New York City during COVID-19. J Am Med Inform Assoc.

[14] Narcisse M, Andersen JA, Felix HC, Hayes CJ, Eswaran H, McElfish PA (2022). Factors associated with telehealth use among adults in the United States: findings from the 2020 National Health Interview Survey. J Telemed Telecare.

[15] Lee S, Black D, Held ML (2019). Factors associated with telehealth service utilization among rural populations. J Health Care Poor Underserved.

[16] Oguz T (2018). Update on racial disparities in access to healthcare: an application of nonlinear decomposition techniques*. Soc Sci Q.

[17] Ghaddar S, Vatcheva KP, Alvarado SG, Mykyta L (2020). Understanding the intention to use telehealth services in underserved Hispanic border communities: cross-sectional study. J Med Internet Res.

[18] (2021). Internet/Broadband Fact Sheet. Pew Research Center.

[19] Lee H, Hodgkin D, Johnson MP, Porell FW (2021). Medicaid expansion and racial and ethnic disparities in access to health care: applying the National Academy of Medicine definition of health care disparities. Inquiry.

[20] Sachs J, Graven P, Gold J, Kassakian S (2021). Disparities in telephone and video telehealth engagement during the COVID-19 pandemic. JAMIA Open.

[21] Dowler S, Crosbie K, Thompson S, Drucker E, Jackson C (2021). Telemedicine utilization trends during the COVID-19 public health emergency. N C Med J.

[22] Jaffe DH, Lee L, Huynh S, Haskell TP (2020). Health inequalities in the use of telehealth in the United States in the lens of COVID-19. Popul Health Manag.

[23] Pierce M, Hope H, Ford T, Hatch S, Hotopf M, John A, Kontopantelis E, Webb R, Wessely S, McManus S, Abel KM (2020). Mental health before and during the COVID-19 pandemic: a longitudinal probability sample survey of the UK population. Lancet Psychiat.

[24] Stevens JP, Mechanic O, Markson L, O'Donoghue A, Kimball AB (2021). Telehealth use by age and race at a single academic medical center during the COVID-19 pandemic: retrospective cohort study. J Med Internet Res.

[25] Anderson M, Perrin A (2017). 1. Technology use among seniors. Pew Research Center.

[26] Pierce RP, Stevermer JJ (2020). Disparities in the use of telehealth at the onset of the COVID-19 public health emergency. J Telemed Telecare.

[27] Wegermann K, Wilder JM, Parish A, Niedzwiecki D, Gellad ZF, Muir AJ, Patel YA (2022). Racial and socioeconomic disparities in utilization of telehealth in patients with liver disease during COVID-19. Dig Dis Sci.

[28] Darrat I, Tam S, Boulis M, Williams AM (2021). Socioeconomic disparities in patient use of telehealth during the coronavirus disease 2019 surge. JAMA Otolaryngol Head Neck Surg.

[29] The Lancet Public Health (2020). Education: a neglected social determinant of health. Lancet Public Health.

[30] Barry K, McCarthy M, Melikian G, Almeida-Monroe V, Leonard M, De Groot AS (2020). Responding to COVID-19 in an uninsured Hispanic/Latino community: testing, education and telehealth at a free clinic in Providence. R I Med J (2013).

[31] Clare CA (2021). Telehealth and the digital divide as a social determinant of health during the COVID-19 pandemic. Netw Model Anal Health Inform Bioinform.

[32] Social Determinants of Health. U.S. Department of Health and Human Services.

[33] Singh AK, Gillies CL, Singh R, Singh A, Chudasama Y, Coles B, Seidu S, Zaccardi F, Davies MJ, Khunti K (2020). Prevalence of co-morbidities and their association with mortality in patients with COVID-19: a systematic review and meta-analysis. Diabetes Obes Metab.

[34] Gumà Jordi, Solé-Auró Aïda, Arpino B (2019). Examining social determinants of health: the role of education, household arrangements and country groups by gender. BMC Public Health.

[35] Kämpfen F, Maurer J (2018). Does education help “old dogs” learn “new tricks”? The lasting impact of early-life education on technology use among older adults. Research Policy.

[36] Regmi K, Mudyarabikwa O (2020). A systematic review of the factors - barriers and enablers - affecting the implementation of clinical commissioning policy to reduce health inequalities in the National Health Service (NHS), UK. Public Health.

[37] Salihu H (2014). Socio-ecological model as a framework for overcoming barriers and challenges in randomized control rrials in minority and underserved communities. Int J MCH AIDS.

[38] The COVID-19 Research Database.

